# Urinary liver-type fatty acid-binding protein levels may be associated with the occurrence of acute kidney injury induced by trauma

**DOI:** 10.3389/fmed.2024.1346183

**Published:** 2024-02-23

**Authors:** Ryu Yasuda, Keiko Suzuki, Hideshi Okada, Takuma Ishihara, Toru Minamiyama, Ryo Kamidani, Yuichiro Kitagawa, Tetsuya Fukuta, Kodai Suzuki, Takahito Miyake, Shozo Yoshida, Nobuyuki Tetsuka, Shinji Ogura

**Affiliations:** ^1^Department of Emergency and Disaster Medicine, Gifu University Graduate School of Medicine, Gifu, Japan; ^2^Department of Infection Control, Gifu University Graduate School of Medicine, Gifu, Japan; ^3^Department of Pharmacy, Gifu University Hospital, Gifu, Japan; ^4^Center for One Medicine Innovative Translational Research, Gifu University Institute for Advanced Study, Gifu, Japan; ^5^Innovative and Clinical Research Promotion Center, Gifu University Hospital, Gifu, Japan; ^6^Abuse Prevention Emergency Medicine, Gifu University Graduate School of Medicine, Gifu, Japan

**Keywords:** acute kidney injury, trauma, new biomarkers, liver-type fatty acid-binding protein, semi-quantitation assay kit

## Abstract

**Introduction:**

Acute kidney injury (AKI), with a fatality rate of 8.6%, is one of the most common types of multiorgan failure in the intensive care unit (ICU). Thus, AKI should be diagnosed early, and early interventions should be implemented. Urinary liver-type fatty acid-binding protein (L-FABP) could aid in the diagnosis of AKI.

**Methods:**

In this prospective, single-center, observational study, we included 100 patients with trauma. Urinary L-FABP levels were measured using a semi-quantitative rapid assay kit 6 and 12 h after injury. Negative, weakly positive, and strongly positive urinary L-FABP levels were examined using two protocols. Using protocol 1, measurements were performed at 6 h after injury negative levels were considered “negative,” and weakly positive and strongly positive levels were considered “positive.” Using protocol 2, strongly positive levels at 6 h after injury were considered “positive,” and negative or weakly positive levels at 6 h after injury were considered “positive” if they were weakly positive or positive at 12 h after injury.

**Results:**

Fifteen patients were diagnosed with AKI. Using protocol 1, the odds ratio (OR) was 20.55 (*p* = 0.001) after adjustment for the injury severity score (ISS), contrast media use, and shock index. When the L-FABP levels at 6 and 12 h were similarly adjusted for those three factors, the OR was 18.24 (*p* < 0.001). The difference in ORs for protocols 1 and 2 was 1.619 (*p* = 0.04).

**Discussion:**

Associations between urinary L-FABP and AKI can be examined more precisely by performing measurements at 6 and 12 h after injury than only one time at 6 h.

## 1 Introduction

Acute kidney injury (AKI) is one of the most common types of multiorgan failure in the intensive care unit (ICU), with a reported fatality rate of 8.6% (1). Therefore, to improve its prognosis, AKI should be diagnosed early, and early interventions should be implemented (1). However, the current diagnostic criteria for AKI, such as an increased serum creatinine level and decreased urine output, include changes that occur as a result of renal injury and may delay intervention; therefore, new biomarkers have been proposed as potentially useful tools for its diagnosis (2, 3). However, which biomarkers should be used and when they should be used have not yet been established. Urinary liver-type fatty acid-binding protein (L-FABP) is expressed in the renal proximal tubules, and its increased expression attributable to ischemia or oxidative stress increases urinary excretion, suggesting that it may be a marker for diagnosing AKI. L-FABP levels are increased early after cardiovascular surgery for patients with AKI (3). Recently, a semiquantitative evaluation kit for urinary L-FABP levels that can be easily performed at the bedside has been developed and reported to be useful in predicting the development of AKI (4, 5). However, many of its target patients have endogenous diseases or developed such after surgery (4, 5), and AKI occurs in approximately 20% of patients even when urinary L-FABP is negative, making this study a sensitivity issue (4). One possible reason for this issue is L-FABP testing is performed after ICU admission, which includes many endogenous diseases for which time since disease onset is difficult to estimate.

A previous meta-analysis reported a 13% complication rate of acute kidney injury in moderate trauma with a median ISS of 14 (6). In addition, trauma often has a more definite onset time than endogenous disease, making it easier to examine the association between the development of AKI and L-FABP levels.

In this study, we investigated whether a urinary L-FABP semiquantitative kit could predict the subsequent onset of AKI in trauma patients 6 and 12 h after trauma injury.

## 2 Materials and methods

### 2.1 Patients

From October 2019 to February 2020, trauma patients 20 years of age or older who were transported directly or by referral and admitted to the ICU of Gifu University Hospital (Gifu, Gifu Prefecture) were included in this study. At our hospital, an arterial access is placed in trauma patients who are deemed to require hemodynamic monitoring upon admission, and a urethral catheter is inserted when urine output measurement is deemed necessary. Therefore, trauma patients who did not require these insertions were considered to have mild trauma and very unlikely to develop AKI; therefore, patients without an implanted intra-arterial pressure line or urethral balloon were excluded from the study. In addition, patients in which more than 6 h had elapsed at the time of transport to our hospital and patients in which specimens could not be collected during emergency surgery or other procedures were excluded from the study. Furthermore, patients of early death in the emergency room, or patients in which the attending physician expected early death, even after admission, patients who could not give study consent, patients with chronic hemodialysis, and patients undergoing renal replacement therapy within 24 h of admission or renal transplantation were also excluded.

### 2.2 Ethics approval and consent to participate

This study conformed to the principles outlined in the Declaration of Helsinki (7). Ethical approval was obtained from the medical ethics committee of Gifu University Graduate School of Medicine, Gifu, Japan (institutional review board approval no. 2018-173). Patients provided written informed consent prior to all study-related procedures. Before initiation, the study was registered with the UMIN Clinical Trials Registry (registry number: UMIN 000038306).

### 2.3 Data collection and study design

This was a single-center, prospective, observational study conducted at Gifu University Hospital, Gifu, Japan. On admission to the ICU, the following clinical variables were evaluated: age, sex, body mass index, presence of chronic diseases, international injury severity score (ISS), shock index (within 6 h of injury), use of contrast media within 6 h of injury acute surgery within 24 h of injury, blood infusion within 24 h of injury, use of rapid renal replacement therapy, and mortality at 28 days. All laboratory data, except patient attributes, were extracted from the hospital’s electronic medical records.

### 2.4 Measurement of L-FABP levels and other tests

Urinary L-FABP levels in urine collected from urethral catheters were evaluated at 6 and 12 h after injury using the RENAPRO^®^ semi-quantitative rapid assay kit (CMIC Pharmaceutical Services Corporation, 1-1-1, Tokyo, Japan). This kit considers the levels negative when they are less than 12.5 ng/mL, weakly positive when 12.5–100 ng/mL, and strongly positive levels at more than 100 ng/mL.

The investigators performed examinations at 6 and 12 h after injury and used two protocols for each patient. Using protocol 1, measurements were performed at 6 h after injury negative levels were considered “negative” and weakly positive and strongly positive levels were considered “positive.”

Using protocol 2, strongly positive levels at 6 h after injury were considered “positive,” and negative or weakly positive levels at 6 h after injury were considered “positive” if they were weakly positive or positive at 12 h after injury. In contrast, negative and weakly positive levels at 6 h after injury were considered “negative” if they were negative at 12 h after injury.

The protocol and indication criteria for this study were thoroughly communicated to all responsible physicians before study commencement. Patients who met the criteria were admitted to the hospital, and if consent was obtained, they were asked to contact the study coordinator, who stored urine samples at 6 and 12 h post-injury in a designated area. The results were not communicated to the treating physician, who was asked to treat the patient to avoid any bias regarding the diagnosis of AKI or intervention.

Blood and urine samples necessary for systemic management were collected at 6 h after injury. respectively, and the data were recorded. The following clinical data were evaluated: creatinine, urea nitrogen, uric acid, cystatin C, and albumin levels; electrolytes (sodium, potassium, hydrogen chloride, calcium, magnesium, and phosphorus in serum); pH level, base excess, and bicarbonate levels in the arteries; hemoglobin levels; platelet count; fibrinogen in whole blood; and pH and specific gravity of urine.

### 2.5 Outcomes

The primary study outcome was the development of AKI within 7 days. The development of AKI was determined by the baseline serum creatinine level within 7 days of injury according to the Kidney Disease Improving Global Outcomes classification (8). Patients with stage 1 or higher disease were considered to have AKI.

### 2.6 Statistical analysis

Patient characteristics and laboratory data were summarized using the median and interquartile range (IQR) for continuous variables and the number and percentage for categorical variables. The association between AKI and each protocol was confirmed using a multivariate logistic regression analysis. Explanatory variables in the logistic regression model (protocol, ISS, contrast media use within 6 h of injury and shock index) were adjusted. Overfitting was possible because of the large number of explanatory variables relative to the number of AKI cases. Therefore, overfitting was avoided by estimating the shrinkage of the regression coefficients. Specifically, we penalized the estimation of the regression coefficients such that the optimism parameter obtained from the 150 bootstrap validations did not exceed 0.2. An association between the protocol and AKI was recognized if the results of the statistical tests of the shrinkage-estimated regression coefficients of the protocol were significant. Because protocols 1 and 2 were used for each patient (paired data), generalized estimating equations were used for comparisons between protocols. The sensitivity and specificity of protocols 1 and 2 for AKI and their 95% confidence intervals (CIs) were calculated. The two-sided significance level was set at 5%. R version 4.3.1 (The R Project for Statistical Computing)^1^ was used for all statistical analyses.

## 3 Results

### 3.1 Patient characteristics

**FIGURE 1:**
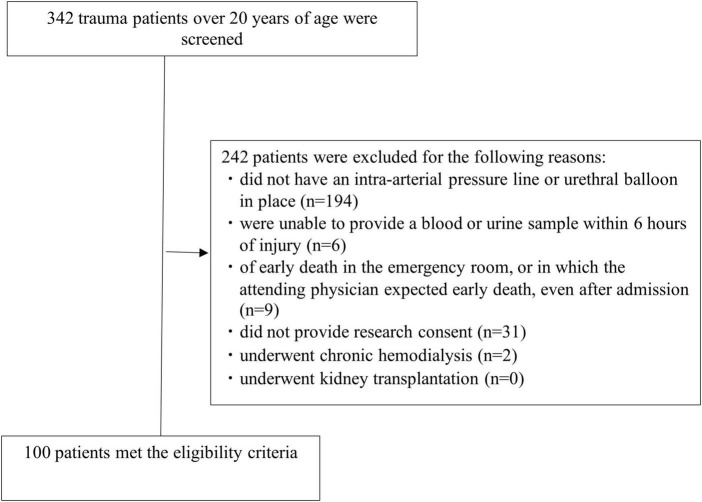
Patients who met the eligibility criteria.

From October 2019 to February 2020, there were 342 trauma patients. Of these, 194 patients without an intra-arterial pressure line or urethral balloon in place, 6 patients who were unable to provide a blood or urine sample within 6 h of injury 9 patients deemed early death in the emergency room, or in which the attending physician expected early death even after admission, 31 patients who were unable to provide research consent, and 2 patients on chronic maintenance dialysis were excluded. None of the 100 patients (67 men and 33 women) dropped out of the study (Figure 1). The patients had a median age of 67.0 years (IQR, 46.0–77.0 years), body mass index of 22.0 (IQR, 19.1–24.2), ISS of 17 (IQR, 9–25), and shock index at 6 hours after injury of 0.74 (IQR, 0.6–0.88). Additionally, 73 patients were administered contrast media within 6 h of injury (Table 1). The clinical data at 6 h after injury are shown in Table 2. Twenty-eight patients underwent acute surgery within 24 h of injury Forty-three patients received blood transfusions within 24 h of injury. Two patients required renal replacement therapy and two patients died within 28 days.

**TABLE 1:** Patient characteristics.

General information	
Age, years, median (IQR)	67 (46–77)
Sex, male, n (%)	67 (67%)
BMI, median (IQR)	22.0 (19.1–24.2)
**Chronic disease (multiple reported per study)**	**n (%)**
Hypertension	25 (25%)
Diabetes	11 (11%)
Stroke	9 (9%)
Coronary artery syndrome	6 (6%)
Hyperuricemia	4 (4%)
Aortic disease	4 (4%)
Urological disorders	2 (2%)
**Associated injuries (multiple reported per study)**	**n (%)**
Head or neck injuries	25 (25%)
Facial injuries	15 (15%)
Chest injuries	53 (53%)
Abdominal or pelvic injuries	32 (32%)
Extremities or pelvic girdle injuries	62 (62%)
External and other trauma injuries	17 (17%)
**Others**	
Injury severity score, median (IQR)	17 (9–25)
Shock index, median (IQR)	0.74 (0.60–0.88)
Use of contract media within 6 h of injury, n (%)	73 (73%)

All data are presented as the median and 25^th^–75^th^ percentile unless otherwise indicated. BMI: body mass index; ICU: intensive care unit; IQR: interquartile range.

**TABLE 2:** Laboratory data at 6 h after ICU admission.

Laboratory data	Median (IQR)
**Cre (mg/dL)**	0.71 (0.58–0.86)
**BUN (mg/dL)**	16.1 (11.45–19.3)
**UA (mg/dL)**	4.9 (3.97–5.9)
**CysC (mg/dL)**	0.71 (0.62–0.87)
**Alb (g/dL)**	3.3 (2.9–3.6)
**Na (mmol/L)**	139 (137–140)
**K (mmol/L)**	4 (3.8–4.3)
**Cl (mmol/L)**	107 (105–108)
**Ca (mmol/L)**	8.3 (8–8.6)
**P (mmol/L)**	3.4 (3–4)
**Mg (mmol/L)**	1.9 (1.8–2)
**pH**	7.39 (7.37–7.43)
**BE**	−0.1 (−2.5–0.8)
**HCO_3_^–^ (mmol/L)**	23.8 (21.8–24.7)
**Hb (g/dL)**	10.1 (9.05–11.6)
**Plt × 10^3^/μL**	157 (101–197)
**FIB (mg/dL)**	195 (162–234.5)
**Urine pH**	6.25 (5.5–7.5)
**Urine gravity**	1.02 (1.01–1.02)

All data are presented as the median and 25^th^–75^th^ percentile unless otherwise indicated. Alb: albumin; BE: base excess; BUN: blood urea nitrogen; Ca: calcium; Cl: hydrogen chloride; Cre: creatinine; FIB: fibrinogen in whole blood; HCO_3_^–^: bicarbonate in the artery; Hb: hemoglobin; ICU: intensive care unit; CysC: cystatin C; K: potassium; Mg: magnesium; Na: sodium; P: phosphorus; Plt: platelets; UA: uric acid.

### 3.2 Urinary L-FABP levels and AKI according to two different protocols

Fifteen patients were diagnosed with AKI. At 6 h after injury, urinary L-FABP levels were strongly positive in 12 patients, weakly positive in 17 patients, and negative in 71 patients. At 12 h after injury, urinary L-FABP levels were strongly positive in 9 patients, weakly positive in 12 patients, and negative in 79 patients.

Using protocol 1, during which L-FABP was semi-quantitatively measured at only one time point (6 h after injury), the odds ratio (OR) was 20.55 (*p* = 0.001) after adjusting for the ISS, contrast media use within 6 h after injury, and shock index. However, when the L-FABP levels at two time points, 6 and 12 h after injury, were similarly adjusted for the ISS, contrast use within 6 h after injury, and shock index, the OR was 18.24 (*p* < 0.001) (Table 3). The difference in ORs for protocols 1 and 2 was 1.619 (*p* = 0.04) (Table 4). The sensitivities and specificities of protocols 1 and 2 for AKI were calculated (Table 5). The sensitivity of protocol 1, which was evaluated at only one time point, 6 h after injury, was higher than that of protocol 2; however, the specificity of protocol 2, which was evaluated at two time points, 6 and 12 h after injury, was higher than that of protocol 1 (Table 6).

**TABLE 3:** Odds ratio for protocol 1.

Variable	Odds ratio	95% LCI	95% UCI	*p*-value
Protocol 1	20.55	3.62	116.76	0.001

LCL: lower confidence limit; UCL: upper confidence limit. The odds ratio was calculated using a logistic regression model with the injury severity score, contrast use within 6 h after injury, and shock index as adjusted factors.

**TABLE 4:** Odds ratio for protocol 2.

Variable	Odds ratio	95% LCI	95% UCI	*p*-value
Protocol 2	18.24	4.21	79.02	< 0.001

LCL: lower confidence limit; UCL: upper confidence limit. The odds ratio was calculated using a logistic regression model with the injury severity score, contrast use within 6 h after injury, and shock index as the adjusted factors.

**TABLE 5:** Difference between odds ratios for protocol 1 and protocol 2.

Variable	Odds ratio	95% LCI	95% UCI	*p*-value
Protocol 2	1.619	1.023	2.562	0.04

LCL: lower confidence limit; UCL: upper confidence limit. The odds ratio was calculated using a logistic regression model with the injury severity score, contrast use within 6 h after injury, and shock index as the adjusted factors.

**TABLE 6:** Sensitivity and specificity of protocol 1 and protocol 2 for acute kidney injury.

**Sensitivity**		
Variable	n = 15	95% CI
**Protocol 1**	12 (80.0%)	51%, 95%
**Protocol 2**	11 (73.3%)	45%, 91%
**Specificity**		
Variable	n = 85	95% CI
**Protocol 1**	68 (81.0%)	71%, 88%
**Protocol 2**	75 (88.2%)	79%, 94%

Data are presented as n (%) unless otherwise indicated. CI: confidence interval.

## 4 Discussion

Recently, several urinary biomarkers have been investigated to determine their use for early detection of and intervention for AKI in critically ill patients. Urinary N-acetyl-β-D-glucosaminidase and β-microglobulin have been used to differentiate whether AKI is renal in origin; however, they are affected by many factors, including chronic kidney disease, and their sensitivity and specificity for the diagnosis of AKI are not high (9). Subsequently, IL-18 (10, 11), kidney molecule-1 (KIM-1) (11, 12), and neutrophil gelatinase-associated lipocalin (NGAL) (11, 13) have been investigated; however, which of these biomarkers should be used and when they should be used have not yet been established. Of these biomarkers, NGAL correlates with AKI severity, but L-FABP was previously reported to be more useful in discriminating between non-AKI and AKI (10). Both can also predict in-hospital mortality, although L-FABP has been reported to be more predictive (14). In terms of pathophysiology, NGAL tends to increase with inflammation and is useful for early diagnosis of sepsis-related AKI, for example. On the other hand, L-FABP increases with decreased renal blood flow, so L-FABP may be suitable for the early diagnosis of trauma-induced AKI, as it can detect decreased renal blood flow at the time of trauma, as in this study. However, other studies have compared L-FABP, NGAL, and KIM-1 and found no difference in predicting the onset of AKI (2). Unlike L-FABP; however, simple test kits, such as those used in this study, are not yet widely available for KIM-1 and NGAL. Thus, L-FABP may be a marker that could be studied in the future as an indicator for early diagnosis of AKI.

L-FABP is a 14-kDa low-molecular-weight protein localized in the cytoplasm of renal proximal tubular cells. Under normal conditions, L-FABP binds to free fatty acids reabsorbed in the tubules and transports them to mitochondria and peroxisomes to promote beta-oxidation, thus contributing to energy production and homeostasis (15).

In tubules with impaired blood flow leading to tubular dysfunction, oxidative stress and increased production of reactive oxygen species convert free fatty acids to highly cytotoxic lipid peroxides, which also increase L-FABP expression. Therefore, it has been suggested that increased urinary L-FABP levels may be useful indicators during the evaluation of AKI associated with tubular dysfunction (10, 16, 17).

Increased L-FABP levels at 4 h after cardiac surgery have been reported (3) and are useful for predicting AKI development with a wide range of diseases in patients who require intensive care (2, 5, 10), those who require emergency care (4), those with sepsis (18), and those with contrast-induced nephropathy (19). Compared to other markers, L-FABP is also associated with the disease prognosis (5, 11). The use of L-FABP to predict chronic kidney disease (20) has been reported, and patients with chronic kidney disease were included in this study. Additionally, L-FABP has been semi-quantitatively evaluated, with negative, weakly positive, and strongly positive levels observed at 15 minutes, and it is useful in general practice (4, 5, 21).

Because no study has examined the association between AKI and urinary L-FABP with trauma using a semi-quantitative kit, it was necessary to establish the timing of L-FABP measurements after trauma. Previous studies have reported that L-FABP can predict the patient prognosis when assessed using a semi-quantitative kit at two time points: at injury and 6 h after injury (4). This two-point measurement method is advantageous because changes can be analyzed. For example, if the first measurement is positive and the second measurement is negative, then tubular ischemia is improved; however, if the first measurement is negative and the second measurement is positive, then the condition is worse. During the present study, L-FABP was measured at two time points; however, unlike previous reports, L-FABP was evaluated at 6 and 12 h after injury (21). This is because bleeding is often uncontrolled immediately after injury, and L-FABP is evaluated after confirming hemostasis by transarterial embolization or other means at the time of arrival at the hospital.

L-FABP, which indicates the blood flow status of the proximal renal tubule, may be an appropriate biomarker reflecting the dynamically changing hemodynamics and acute phase status of systemic lesions. At 6 h after injury a positive result (including a weakly positive result) indicated that the blood flow in the proximal renal tubule was impaired. In contrast, a weakly positive result at 6 h after injury and a negative result at 12 h may indicate that blood flow has improved; however, a weakly positive or positive result at 12 h may indicate that blood flow in the renal tubules is still impaired. Based on these results, it is considered appropriate to perform repeat testing at 12 h if the level is negative or weakly positive at 6 h.

This study had some limitations. First, it was a single-center study. Second, although urinary L-FABP levels are increased in patients with abdominal trauma (22), the number of patients was insufficient to allow a subgroup analysis or strict confounding adjustments. Third, interventions prior to arrival at the hospital were variable. We included both patients who were directly transported from the trauma scene to our hospital as well as those who were transported to our hospital after being admitted to another hospital and receiving interventions such as intravenous fluid infusion. Finally, comparisons with other urinary markers were not performed.

## 5 Conclusion

The results of a semi-quantitative kit used to measure urinary L-FABP were associated with the development of AKI after trauma. Although it was suggested that this association may be more accurately examined by performing measurements at 6 h and again at 12 h after injury, multiple markers should be examined simultaneously to allow a more comprehensive investigation of markers that allow for the early diagnosis of AKI.

## Data availability statement

The raw data supporting the conclusions of this article will be made available by the authors, without undue reservation.

## Ethics statement

The studies involving humans were approved by the medical ethics committee of Gifu University Graduate School of Medicine, Gifu, Japan (institutional review board approval no. 2018-173). The studies were conducted in accordance with the local legislation and institutional requirements. The participants provided their written informed consent to participate in this study.

## Author contributions

RY: Conceptualization, Writing – original draft. KeS: Conceptualization, Data curation, Writing – original draft. HO: Conceptualization, Writing – review and editing. TI: Formal analysis, Writing – review and editing. ToM: Data curation, Writing – review and editing. RK: Data curation, Writing – review and editing. YK: Data curation, Writing – review and editing. TF: Investigation, Writing – review and editing. KoS: Investigation, Writing – review and editing. TaM: Investigation, Writing – review and editing. SY: Supervision, Writing – review and editing. NT: Data curation, Writing – review and editing. SO: Supervision, Writing – review and editing.

## Footnotes

1
www.r-project.org
